# Mannosylated Polyrotaxanes for Increasing Cellular Uptake Efficiency in Macrophages through Receptor-Mediated Endocytosis

**DOI:** 10.3390/molecules24030439

**Published:** 2019-01-26

**Authors:** Kai Shibaguchi, Atsushi Tamura, Masahiko Terauchi, Mitsuaki Matsumura, Hiroyuki Miura, Nobuhiko Yui

**Affiliations:** 1 Department of Restorative Sciences, Graduate School of Medical and Dental Sciences, Tokyo Medical and Dental University (TMDU), 1-5-45 Yushima, Bunkyo, Tokyo 113-8549, Japan; k.shibaguchi.fpro@tmd.ac.jp (K.S.); mmdent@mvd.biglobe.ne.jp (M.M.); h.miura.fpro@tmd.ac.jp (H.M.); 2 Department of Organic Biomaterials, Institute of Biomaterials and Bioengineering, Tokyo Medical and Dental University (TMDU), 2-3-10 Kanda-Surugadai, Chiyoda, Tokyo 101-0062, Japan; terauchi.org@tmd.ac.jp (M.T.); yui.org@tmd.ac.jp (N.Y.)

**Keywords:** polyrotaxane, cyclodextrin, macrophage, mannose, macrophage mannose receptor

## Abstract

Macrophages play an important role in the regulation of inflammation and immune response as well as the pathogenesis of chronic inflammatory diseases and cancer. Therefore, targeted delivery of therapeutic reagents to macrophages is an effective method for treatment and diagnosis. We previously examined the therapeutic applications of polyrotaxanes (PRXs) comprised of multiple cyclodextrins (CDs) threaded on a polymer chain and capped with bulky stopper molecules. In the present study, we designed an α-d-mannose-modified α-CD/poly(ethylene glycol)-based PRX (Man-PRX). The intracellular uptake of Man-PRX through the interaction with macrophage mannose receptor (MMR) in macrophage-like RAW264.7 cells was examined. Intracellular Man-PRX uptake was observed in MMR-positive RAW264.7 cells but was negligible in MMR-negative NIH/3T3 cells. In addition, the intracellular Man-PRX uptake in RAW264.7 cells was significantly inhibited in the presence of free α-d-mannose and an anti-MMR antibody, which suggests that MMR is involved in the intracellular uptake of Man-PRX. Moreover, the polarization of RAW264.7 cells affected the Man-PRX internalization efficiency. These results indicate that Man-PRX is an effective candidate for selective targeting of macrophages through a specific interaction with the MMR.

## 1. Introduction

Polyrotaxanes (PRXs) are representative supramolecular polymers comprising multiple cyclodextrins (CDs) threaded along a polymer chain capped with bulky stopper molecules [1,2,3,4]. One of the unique characteristics of PRXs is the ability of the mechanically interlocked CDs to freely slide and rotate along the polymer axle. Therefore, the threaded CDs can be immediately released when the bulky terminal stopper molecules are liberated in response to specific stimuli, such as pH, reduction, and light irradiation [5]. Our group had previously developed such stimuli-cleavable PRXs and investigated their applications in biomaterials and drug delivery [4,5]. We recently reported the development of β-CD-threaded acid-labile PRXs, which can release the β-CDs in response to the acidic pH in late endosomes and lysosomes, through acid-induced liberation of bulky stoppers [6,7,8,9,10,11]. We found that the β-CDs released from the acid-labile PRXs interact with intracellular cholesterols and lipids by forming an inclusion complex, which leads to the modulation of cellular metabolic functions. For example, acid-labile PRXs improve the accumulation of unesterified cholesterol in a cell culture and mouse models of Niemann-Pick type C disease by excreting cholesterols [6,7,8], promoting the excretion of bisretinoids accumulated in a cell culture model of age-related macular degeneration [9], and inducing autophagy, which is a bulk degradation system for intracellular proteins and organelles, through the induction of endoplasmic reticulum-stress [10]. Since these functions are not observed or are less effective with free CDs, acid-labile PRXs can be potentially applied for treating metabolic diseases such as atherosclerosis [12].

Macrophages play an important role in inflammation, immune system responses, and tissue repair [13,14]. Macrophage-induced chronic inflammation is associated with the development of various diseases such as atherosclerosis [15]. Therefore, targeted delivery of PRXs to macrophages is regarded as an effective strategy for preventing and treating various diseases by altering metabolic functions. However, the molecular design of PRXs needs to be improved to achieve macrophage targeting. Macrophages express mannose-binding C-type lectin receptors, such as the macrophage mannose receptor (MMR, also referred to as CD206), which is a 180-kDa transmembrane glycoprotein [16,17]. The extracellular region of the MMR contains an N-terminal cysteine-rich domain, a fibronectin type II domain, and eight carbohydrate recognition domains that specifically bind to oligosaccharides, including mannose, fucose, and *N*-acetylglucosamine [16,17,18]. Therefore, mannose-conjugation is a promising approach for targeting macrophages through specific recognition by the MMR [18,19,20]. To date, various mannose-conjugated proteins, polymers, nanoparticles, liposomes, and imaging probes have been developed for treating and diagnosing various diseases, such as cancer, infectious diseases, and metabolic diseases [21,22,23,24,25]. 

Therefore, we designed mannose-modified PRXs and investigated their ability to modulate anti-inflammatory and proinflammatory cytokine gene expression [26]. However, to date, the ability of mannose-modified PRXs to interact with the MMR and their intracellular uptake in macrophages have not been clarified. In the present study, we synthesized α-d-mannose-modified PRX (Man-PRX) and examined its intracellular uptake in macrophage-like RAW264.7 cells in comparison with water-soluble 2-(2-hydroxyethoxy) ethyl (HEE) carbamate-modified PRX (HEE-PRX), which was used in our previous studies [6,7,8,9]. In addition, the involvement of MMR in the intracellular uptake of Man-PRX in RAW264.7 cells was investigated using competitive inhibitors or stimulating macrophage polarization to alter the expression level of MMR.

## 2. Results and Discussion

### 2.1. Synthesis and Characterization of Man-PRX

**Figure 1:**
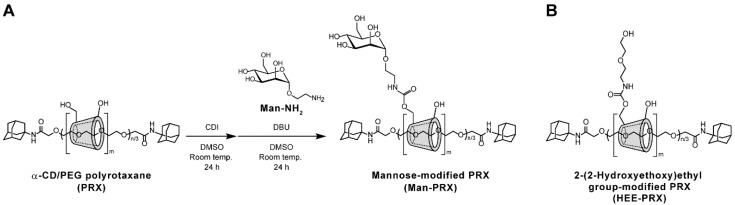
(**A**) Scheme for synthesizing α-d-mannose-modified α-CD/PEG-based PRX (Man-PRX) and (**B**) chemical structure of 2-(2-hydroxyethoxy)ethyl carbamate-modified PRX (HEE-PRX), where *n* and *m* denote the polymerization degree of the PEG axle and the number of threaded α-CDs, respectively.

To date, mannose-modified α-CD/poly(ethylene glycol) (PEG)-based PRXs or pseudo-PRXs have been synthesized using a click reaction between azidated PRXs and propargyl mannose [26,27,28,29]. Although the click reaction is highly efficient for introducing functional molecules on PRXs, it requires the introduction of azide or alkyne groups. In the present study, α-d-mannose was chemically modified on the threaded α-CDs in PRX by activating the hydroxyl groups of the threaded α-CDs with 1,1′-carbonyldiimidazole (CDI) in dimethyl sulfoxide (DMSO), followed by a reaction with 2-aminoethyl α-d-mannopyranoside (Man-NH_2_) in the presence of 1,8-diazabicyclo [5.4.0]undec-7-ene (DBU) (Figure 1A) [30,31]. PRX containing α-CD as a cyclic molecule, PEG (*M*_n_: 9800, polymerization degree: 222) as an axle polymer, and adamantyl groups as stopper molecules, was synthesized for the modification of Man-NH_2_. The number of threaded α-CDs and *M*_n_ of the precursor PRX were determined to be 26.7 (the threading percentage of α-CDs in PRX was 24.7%, assuming that one α-CD molecule forms an inclusion complex with two ethylene glycol units in PEG) and 36,100, respectively.

**Figure 2:**
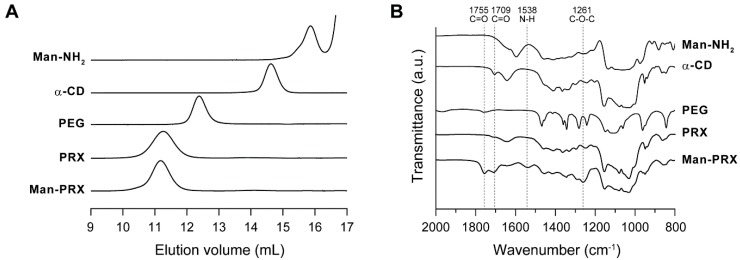
(**A**) SEC charts of Man-NH_2_, α-CD, axle PEG (biscarboxy-PEG, *M*_n_: 9800), unmodified PRX, and Man-PRX in DMSO containing 10 mM LiBr at 60 °C. (**B**) FT-IR spectra of Man-NH_2_, α-CD, axle PEG, unmodified PRX, and Man-PRX.

The produced Man-PRX was characterized using size exclusion chromatography (SEC), Fourier transform infrared (FT-IR) spectroscopy, ^1^H nuclear magnetic resonance (NMR), and ^13^C NMR. In the SEC charts of Man-PRX, a single unimodal peak was observed (*M*_w_/*M*_n_ = 1.15), whereas negligible peaks were detected for free Man-NH_2_, α-CDs, and PEG. This indicates that these impurities were completely removed during the purification (Figure 2A). In the FT-IR spectrum of Man-PRX, new peaks were observed at 1261, 1538, 1709, and 1755 cm^−1^, in comparison with unmodified PRX (Figure 2B). These peaks are attributed to the streching vibration of C–O–C (1261 cm^−1^), the bending vibration of N–H (1538 cm^−1^), and the streching vibration of hydrogen bonded and free C=O (1709 and 1755cm^−1^) [32]. These results strongly suggest that Man-NH_2_ was covalently attached to the hydroxy groups of the threaded α-CDs via carbamate linkages.

**Figure 3:**
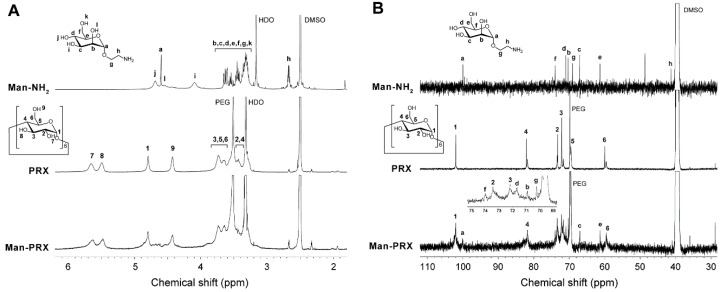
(**A**) ^1^H NMR and (**B**) ^13^C NMR spectra of Man-NH_2_, unmodified PRX, and Man-PRX in DMSO-*d*_6_ at 25 °C.

In the ^1^H NMR spectrum of Man-PRX, the peaks of α-d-mannose moieties appeared around 4.5–4.7 ppm. However, most of the peaks of Man-NH_2_ (3.2–3.8 ppm) overlapped with those of threaded α-CDs in PRX (Figure 3A). ^13^C NMR spectrum of Man-PRX clearly showed the new peaks of α-d-mannose moieties at 61.2, 67.0, 70.2, 70.8, 71.6, 73.9, and 102.0 ppm (Figure 3B). These results indicate the successful modification of α-d-mannose onto PRX. The number of modified α-d-mannose molecules in PRX was determined to be 56.5 by comparing the integral ratio between 4.8 ppm (H_1_ proton of threaded α-CD) and 3 to 4 ppm (-CH_2_-CH_2_-O- of PEG, H_2_, H_3_, H_4_, H_5_, and H_6_ protons of threaded α-CD, H_2_, H_3_, H_4_, H_5,_ H_6,_ -O_6_H, and -CH_2_-CH_2_-NH-C(=O)-O-protons of α-d-mannose) in the ^1^H NMR spectrum in D_2_O (data not shown). As a result, the number of modified α-d-mannose molecules on PRX and *M*_n_ of Man-PRX were determined to be 56.5 and 49,800, respectively. The solubility of PRX in aqueous media was remarkably improved by modifying α-d-mannose, which results in a solubility of >130 mg/mL, even though α-CD/PEG-based PRXs are generally insoluble in aqueous media because of the formation of intermolecular hydrogen bonds [1,33]. As a control, water-soluble 2-(2-hydroxyethoxy)ethyl carbamate-modified PRX (HEE-PRX), which was designed to solubilize PRXs in water in our previous studies [6,7,8,9,30,31], was synthesized using the same PRX (Figure 1B). The number of modified HEE groups in PRX and the *M*_n_ of HEE-PRX were determined to be 115 and 51,200, respectively. HEE-PRX also showed an excellent solubility in aqueous media (>150 mg/mL).

### 2.2. Analysis of the Binding of Man-PRX to the MMR

**Figure 4:**
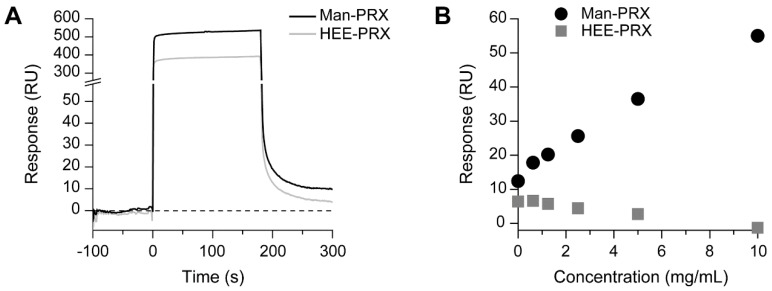
(**A**) SPR sensor grams of Man-PRX (5 mg/mL) and HEE-PRX (5 mg/mL) on the mouse MMR-immobilized sensor chip surfaces. (**B**) Relationship between PRX concentration and the SPR response. The circles and squares in the plot indicate Man-PRX and HEE-PRX, respectively.

The binding affinity of Man-PRX to the MMR was assessed by performing surface plasmon resonance (SPR). The recombinant mouse MMR was immobilized on the SPR sensor chips, and the binding level of Man-PRX to the sensor chips was determined. Figure 4A shows the sensor grams of Man-PRX and HEE-PRX on the MMR-immobilized sensor chip surfaces. We observed that Man-PRX-treated sensor chip surfaces yielded a slightly higher signal (response unit, RU) than HEE-PRX-treated sensor chip surfaces after washing at 180 s. The relationship between PRX concentration and the RU value is shown in Figure 4B. We observed that the RU value of the Man-PRX-treated sensor chip surfaces proportionally increased with an increase in the concentration of Man-PRX. However, the RU value of the HEE-PRX-treated sensor chip surfaces was low even at a HEE-PRX concentration of 10 mg/mL. This result suggests that Man-PRX specifically interacts with the MMR while HEE-PRX barely interact with the MMR. The association and dissociation rate constants between Man-PRX and the MMR were *k*_a_ = 1.3 × 10^3^ (M^−1^·s^−1^) and *k*_d_ = 1.4 (s^−1^). The dissociation constant between Man-PRX and the MMR was *K*_D_ = 1.0 × 10^−3^ (M), which is remarkably higher than the value reported in a previous study (10 nM) [18]. In our previous study, the association rate constant was affected by the density of proteins immobilized on the SPR sensor chip and the number of modified mannose molecules on PRX [28]. We concluded that the number of α-d-mannose molecules in Man-PRX or the density of the MMR on the sensor chips was insufficient to show a low dissociation constant.

### 2.3. Intracellular Man-PRX Uptake in RAW264.7 Cells

**Figure 5:**
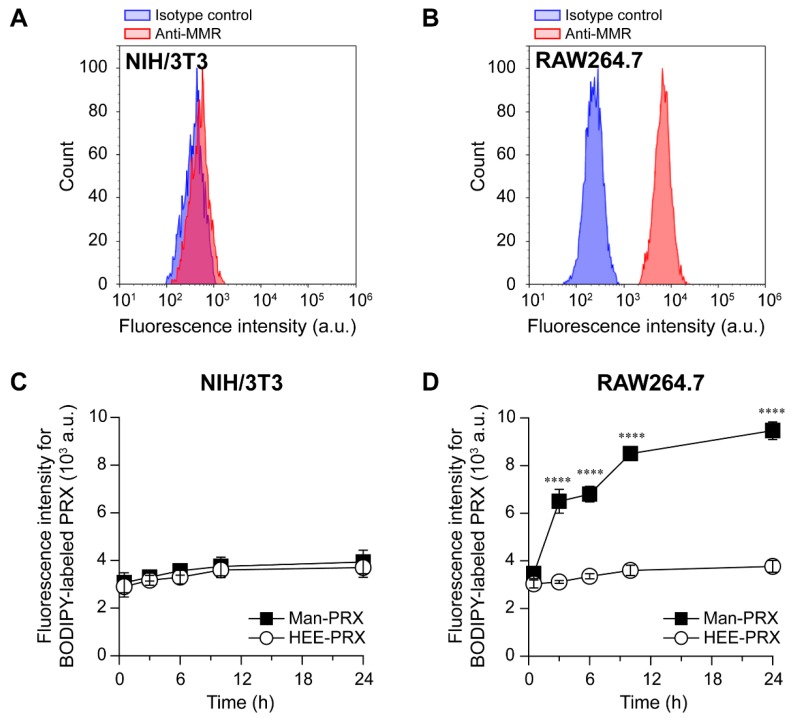
Fluorescence intensities of (**A**) NIH/3T3 and (**B**) RAW264.7 cells treated with the APC-labeled IgG2a,κ isotype control and APC-anti-MMR antibody. The time course of fluorescence intensity of (**C**) NIH/3T3 and (**D**) RAW264.7 cells treated with BODIPY-HEE-PRX (10 μM, open circles) and BODIPY-Man-PRX (10 μM, closed squares). Data are expressed as mean ± standard deviation (n = 3, **** *p* < 0.001).

To clarify the effect of α-d-mannose modification on the intracellular uptake of Man-PRX through the MMR, we assessed the intracellular uptake of Man-PRX in RAW264.7 cells (mouse macrophage-like cells) because they exhibit a high MMR expression [34]. As a control, NIH/3T3 (mouse fibroblasts) cells were used as MMR-negative cells [35]. The flow cytometric analysis of MMR using allophycocyanin (APC)-conjugated anti-MMR antibody revealed that MMR expression was not observed in NIH/3T3 cells, but was significant in RAW264.7 cells (Figure 5A,B). To evaluate the intracellular Man-PRX uptake by flow cytometry and confocal laser scanning microscopy (CLSM), BODIPY FL fluorescent molecules were modified onto Man-PRX (BODIPY-Man-PRX) because of their high molar absorption coefficient, high fluorescence quantum yield, and insensitivity to solvent polarity and pH [36]. BODIPY-labeled HEE-PRX (BODIPY-HEE-PRX) was also prepared as a control. First, we investigated the time-course of intracellular uptake of BODIPY-Man-PRX and BODIPY-HEE-PRX by performing flow cytometry (Figure 5C,D). The intracellular uptake of BODIPY-Man-PRX and BODIPY-HEE-PRX was comparable in the MMR-negative NIH/3T3 cells after 24 h of incubation. In contrast, the intracellular uptake of BODIPY-Man-PRX was significantly higher than that of BODIPY-HEE-PRX in the MMR-positive RAW264.7 cells.

**Figure 6:**
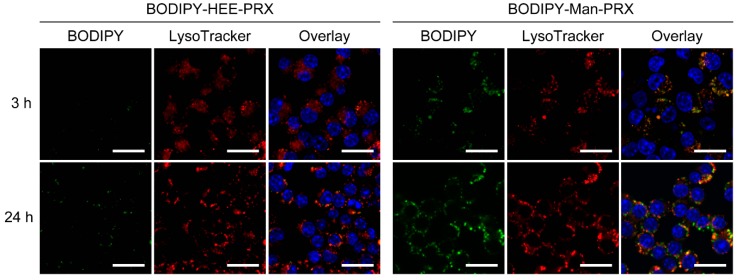
CLSM images of RAW264.7 cells treated with BODIPY-HEE-PRX (10 μM, green, first row) and BODIPY-Man-PRX (10 μM, green, first row) for 3 h and 24 h (scale bars: 20 μm). The cells were stained with Hoechst 33,342 (blue) and LysoTracker Red (red, second row) to visualize the cell nuclei and lysosomes, respectively. The third row depicts overlay images.

Next, we evaluated the intracellular distribution of BODIPY-Man-PRX in the MMR-positive RAW264.7 cells by performing CLSM (Figure 6). To visualize the localization, the cell nuclei and late endosomes/lysosomes were stained with Hoechst 33,342 and LysoTracker Red, respectively. The fluorescence signals of BODIPY-HEE-PRX were not observed after 3 h and 24 h of incubation. In contrast, the fluorescence signals of BODIPY-Man-PRX were clearly observed after 3 h of incubation, which was consistent with the results of flow cytometry. At 3 h of incubation, BODIPY-Man-PRX was found to be co-localized with late endosomes/lysosomes, which indicates that BODIPY-Man-PRX was internalized in RAW264.7 cells through endocytosis. Image analysis showed that the co-localization percentage of BODIPY-Man-PRX with LysoTracker Red was 76.1% after 3 h and 52.5% after 24 h of incubation. This result indicates that Man-PRX was transferred to other organelles or reached the cytoplasm. However, the detailed underlying mechanisms are unclear.

**Figure 7:**
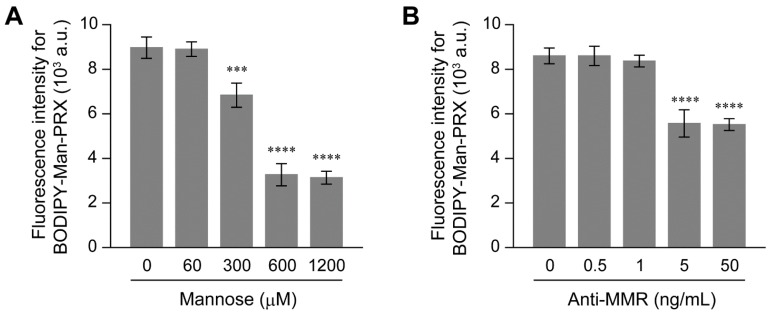
Fluorescence intensity of RAW 264.7 cells pretreated with various concentrations of (**A**) α-d-mannose and (**B**) anti-MMR antibody for 3 h, followed by treatment with BODIPY-Man-PRX for 3 h. The concentration of BODIPY-Man-PRX was 10 μM, which corresponded to 565 μM α-d-mannose molecules modified on PRX. Data are expressed as mean ± standard deviation (n = 3, *** *p* < 0.005 and **** *p* < 0.001).

To verify the involvement of the MMR on the intracellular BODIPY-Man-PRX uptake in RAW264.7 cells, the intracellular BODIPY-Man-PRX uptake was competitively inhibited using free α-d-mannose (Figure 7A) [21]. The intracellular BODIPY-Man-PRX uptake reduced with an increase in the concentration of free α-d-mannose in the culture medium. The significant inhibition of Man-PRX uptake was observed when the free α-d-mannose concentration was higher than the concentration of modified α-d-mannose in Man-PRX. This result suggests that the modified α-d-mannose in Man-PRX plays an essential role in the intracellular uptake of PRXs in MMR-positive RAW264.7 cells. Similarly, RAW264.7 cells were pretreated with anti-MMR antibody to mask the MMR. The intracellular BODIPY-Man-PRX uptake decreased upon pre-treatment with the anti-MMR antibody (Figure 7B). Although the binding sites of mannose and anti-MMR antibody may be different, it was considered that large antibody molecules sterically inhibited the interaction between Man-PRX and MMR on the surface of the cells. According to these results, the MMR was involved in the intracellular uptake of Man-PRX in RAW264.7 cells.

### 2.4. Effect of RAW264.7 Cell Polarization on the Intracellular Man-PRX Uptake

Macrophages perform different functions upon stimulation with various factors. Lipopolysaccharide (LPS) and interferon (IFN)-γ polarize macrophages toward the M1 phenotype [37,38], which are characterized by the secretion of proinflammatory cytokines and the expression of major histocompatibility complex class II and co-stimulatory molecules, in order to initiate and sustain inflammation. In contrast, interleukin (IL)-4 polarizes macrophages toward the M2 phenotype, which is characterized by the secretion of anti-inflammatory cytokines, in order to resolve inflammation and initiatetissue repair [37,38]. Note that the MMR expression level was increased in M2 macrophages. Therefore, it was concluded that the intracellular Man-PRX uptake might be affected by macrophage polarization. 

**Figure 8:**
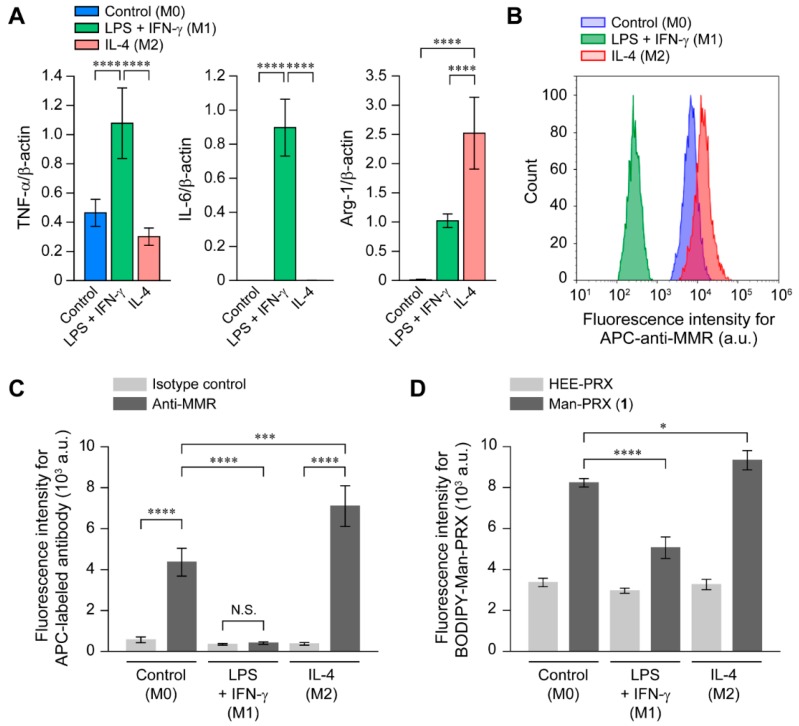
(**A**) mRNA expression levels of TNF-α, IL-6, and Arg-1 in RAW 264.7 cells polarized with LPS (100 ng/mL) + IFN-γ (100 ng/mL) (M1 polarization), or IL-4 (400 ng/mL) (M2 polarization), for 24 h. The phenotype of untreated RAW264.7 cells was denoted as M0. The expression levels of each gene were normalized by the expression level of the β-actin gene (n = 4). (**B**,**C**) The fluorescence intensity of RAW264.7 cells polarized with LPS + IFN-γ or IL-4 for 24 h, followed by treatment with the APC-labeled IgG2a,κ isotype control and APC-anti-MMR antibody (n = 3). (**D**) The fluorescence intensity of RAW264.7 cells polarized with LPS + IFN-γ or IL-4 for 24 h was followed by treatment with BODIPY-HEE-PRX (10 μM) and BODIPY-Man-PRX (10 μM) for 3 h. Data are expressed as mean ± standard deviation (n = 3, * *p* < 0.05, *** *p* < 0.005, and **** *p* < 0.001. N.S. indicates no significance).

To investigate the effect of RAW264.7 cell polarization on the intracellular Man-PRX uptake, RAW264.7 cells were stimulated with LPS and IFN-γ to polarize them toward the M1 phenotype, or with IL-4 to polarize them toward the M2 phenotype. In the present study, the phenotype of the untreated cells was denoted as M0. The phenotype of RAW264.7 cells was characterized by evaluating the gene expression levels of the tumor necrosis factor (TNF)-α and IL-6 as a marker of M1 polarization, and arginase-1 (Arg-1) as a marker of M2 polarization (Figure 8A) [37,39]. RAW264.7 cells treated with LPS and IFN-γ showed significantly increased TNF-α and IL-6 expression levels while those treated with IL-4 showed significantly increased Arg-1 expression levels, when compared with untreated cells. Next, the effect of macrophage polarization on the MMR expression level was investigated (Figure 8B,C). The MMR expression level was upregulated in M2 macrophages, but significantly downregulated in M1 macrophages, compared with the M0 macrophages, which is consistent with previous reports [40,41]. These results indicate a successful polarization of RAW264.7 cells in the M1 and M2 phenotypes.

The effect of RAW264.7 cell polarization on the intracellular uptake of Man-PRX and HEE-PRX was evaluated using flow cytometry (Figure 8D). In addition, we observed that, compared with the M0 macrophages, the intracellular BODIPY-Man-PRX uptake increased in M2 macrophages and decreased in M1 macrophages. However, the intracellular BODIPY-HEE-PRX uptake was similar across all three macrophage polarization states. This result suggests that the MMR expression level is an important factor for determining the intracellular Man-PRX uptake, and that Man-PRX is preferentially internalized in M2 macrophages via MMR-mediated endocytosis.

## 3. Materials and Methods

### 3.1. Materials 

PRX containing α-CD as a cyclic molecule, PEG (*M*_n_: 9800, polymerization degree: 222) as an axle polymer, and adamantyl groups as stopper molecules, was synthesized in accordance with a previous study [42]. In addition, Man-NH_2_ and HEE-PRX (*M*_n_ of PEG axle: 9800, number of threaded α-CDs: 26.7, number of HEE groups modified on PRX: 115, *M*_n_: 51,200) were also synthesized in accordance with previous studies [30,31,43]. 

### 3.2. Instrumentation

^1^H NMR and ^13^C NMR spectra were recorded in DMSO-*d*_6_ at 25 °C, using a Bruker Avance III 400 MHz spectrometer (Bruker BioSpin, Rheinstetten, Germany). Chemical shifts in ^1^H NMR and ^13^C NMR spectra were referenced using tetramethylsilane (0 ppm) and DMSO (39.5 ppm), respectively. SEC was performed using Prominence-i LC-2030 Plus (Shimadzu, Kyoto, Japan) equipped with an RID-20A refractive index detector (Shimadzu) and a combination of TSKgel α-4000 and α-2500 columns (300 mm length, 7.8 mm internal diameter) (Tosoh, Tokyo, Japan). Sample solutions were injected into the system and then eluted with DMSO containing 10 mM LiBr at a flow rate of 0.35 mL/min at 60 °C. The polydispersity index (*M*_w_/*M*_n_) was calculated using a calibration curve of standard PEG (Agilent Technologies, Wilmington, DE, USA).

### 3.3. Synthesis of Man-PRX

PRX (*M*_n_ of the PEG axle: 9800, number of threaded α-CDs: 26.7, *M*_n_ of PRX: 36,100) (300 mg, 8.31 μmol PRX, 222 μmol threaded α-CDs in PRX) and CDI (358 mg, 2.21 mmol, Merck, Darmstadt, Germany) were dissolved in dehydrated DMSO (30 mL, Kanto Chemicals, Tokyo, Japan) under a nitrogen atmosphere, and. the solution was stirred for 24 h at room temperature. DBU (1.32 mL, 8.88 mmol, Merck) and Man-NH_2_ (1.97 g, 8.82 mmol) were added to the reaction mixture, and the solution was stirred for 24 h at room temperature. The resulting solution was purified by dialysis against water for 3 days by using Spectra/Por 4 (molecular weight cut-off [MWCO]: 12,000–14,000, Spectrum Laboratories, Rancho Dominguez, CA, USA). The recovered solution was freeze-dried to yield Man-PRX (331 mg 80.0% yield). ^1^H NMR (400 MHz, DMSO-*d*_6_): δ = 1.62 (m, adamantyl group), 1.93 (m, adamantyl group), 2.03 (m, adamantyl group), 3.06–4.01 (m, -CH_2_-CH_2_-O- of PEG, H_2_, H_3_, H_4_, H_5_, and H_6_ protons of α-CD, and H_2_, H_3_, H_4_, H_5,_ H_6,_ -O_6_H, and -CH_2_-CH_2_-NH-C(=O)-O- protons of α-d-mannose), 4.42 (m, -O_6_H proton of α-CD), 4.59–4.74 (m, H_1_, -O_2_H, and -O_4_H protons of α-d-mannose), 4.79 (m, H_1_ proton of α-CD), 5.48 (m, -O_3_H proton of α-CD), 5.63 (m, -O_2_H proton of α-CD). ^13^C NMR (100 MHz, DMSO-*d*_6_): δ = 59.5 (C_6_ of α-CD), 61.2 (C_6_ of α-d-mannose), 67.0 (C_3_ of α-d-mannose), 69.7 (-**C**H_2_-**C**H_2_-O- of PEG and C_5_ of α-CD), 70.2 (-**C**H_2_-CH_2_-NH-C(=O)-O- protons of α-d-mannose), 70.8 (C_2_ of α-d-mannose), 71.6 (C_4_ of α-d-mannose), 72.1 (C_3_ of α-CD), 73.4 (C_2_ of α-CD), 73.9 (C_5_ of α-d-mannose), 81.7 (C_4_ of α-CD), 100.0 (C_1_ of α-CD), and 102.0 (C_1_ of α-d-mannose).

### 3.4. Synthesis of BODIPY-Labeled PRX

Man-PRX (100 mg, 2.01 μmol PRX, 53.6 μmol threaded α-CDs in PRX) and CDI (38.2 mg, 236 μmol) were dissolved in dehydrated DMSO (5 mL) under a nitrogen atmosphere and the solution was stirred for 6 h at room temperature. 4,4-Difluoro-5,7-dimethyl-4-bora-3a,4a-diaza-s-indacene-3-propionyl ethylenediamine hydrochloride (BODIPY FL EDA; Thermo Fisher Scientific, Waltham, MA, USA) (1.02 mg, 2.75 μmol) was added to the reaction mixture, and the solution was stirred for 24 h at room temperature, with protection from light. After the reaction, the resulting polymer was purified by dialysis against water for 3 days using Spectra/Por 4 (MWCO: 12,000–14,000), and the recovered solution was freeze-dried to yield BODIPY-labeled Man-PRX (BODIPY-Man-PRX) (99.4 mg). The number of BODIPY molecules modified onto Man-PRX was determined by measuring the absorbance at 503 nm (one BODIPY molecule was modified onto 6.3 Man-PRX molecules). BODIPY-labeled HEE-PRX (BODIPY-HEE-PRX) was synthesized and characterized in the same manner. In subsequent experiments, unlabeled and BODIPY-labeled PRXs were mixed to adjust the fluorescence intensities of BODIPY-Man-PRX and BODIPY-HEE-PRX.

### 3.5. Analysis of the Binding between Man-PRX and the MMR by SPR

When performing SPR, the MMR was immobilized on a sensor chip using a previously described protocol [44,45]. Briefly, CM5 sensor chips (GE Healthcare, Chicago, IL, USA), with a carboxymethylated dextran-immobilized gold surface, were treated with a buffer solution containing 10 mM 2-[4-(2-hydroxyethyl)piperazin-1-yl]ethanesulfonic acid (HEPES), 200 mM 1-ethyl-3-(3-dimethylaminopropyl)carbodiimide hydrochloride, and 50 mM N-hydroxysuccinimide at a flow rate of 5 μL/min, in a Biacore X100 instrument (GE Healthcare). Next, recombinant mouse MMR (R & D Systems, Minneapolis, MN, USA) (15.4 μg/mL) was immobilized on the activated CM5 sensor chips in 10 mM sodium acetate buffer (pH 5.5) with a flow rate of 5 μL/min at 25 °C for 1080 s. The response unit (RU) of the MMR in this condition was approximately 6,000 RU. The binding affinity of Man-PRX and HEE-PRX toward the MMR-immobilized sensor chip surfaces was analyzed by dissolving different concentrations of Man-PRX and HEE-PRX in a buffer solution containing 10 mM HEPES, 150 mM NaCl, 3 mM EDTA, 0.005% surfactant P20, 1 mM CaCl_2_, and 1 mM MgCl_2_, which was followed by treatment of the MMR-immobilized sensor chip surfaces with the PRX solutions at a flow rate of 30 μL/min at 25 °C for 180 s. The calibrated sensorgrams were fitted to a Langmuir 1:1 binding model, and the kinetic parameters were determined using Biacore X100 Evaluation Software (version 2.0.1, GE Healthcare) [28].

### 3.6. Cell Culture 

RAW264.7 cells, a mouse macrophage-like cell line, and NIH/3T3 cells, a mouse embryonic fibroblast cell line, were obtained from the American Type Culture Collection (ATCC, Manassas, VA, USA) and the Japanese Collection of Research Bioresources (JCRB, Osaka, Japan), respectively. Both the cell lines were cultured in Dulbecco’s modified Eagle’s medium (DMEM) (Fujifilm Wako Pure Chemical, Osaka, Japan) supplemented with 10% fetal bovine serum (Gibco, Grand Island, NY, USA), 100 units/mL penicillin (Fujifilm Wako Pure Chemical), and 100 µg/mL streptomycin (Fujifilm Wako Pure Chemical) in 5% CO_2_ at 37 °C.

### 3.7. Expression Level of the MMR 

NIH/3T3 or RAW264.7 cells cultured in 24-well plates (Thermo Fisher Scientific) were harvested and collected by centrifugation at 350× *g* and 4 °C for 5 min. The RAW264.7 cells were treated with TruStain FcX (anti-mouse CD16/32 antibody, clone: 93, BioLegend, San Diego, CA, USA) for 15 min at 4 °C to block FCγ receptors III and II. Next, the cells were stained with APC-labeled anti-mouse MMR (APC-MMR) antibody (clone: C068C2, Biolegend) or APC-labeled mouse IgG2a,κ isotype control antibodies (clone: MOPC-173, Biolegend) for 10 min at 4 °C. The cells were then collected by centrifugation at 350× *g* and 4 °C for 5 min, washed with phosphate buffered saline containing 0.1% bovine serum albumin, and passed through a 35-μm cell strainer (Corning, Corning, NY, USA). The fluorescence intensity of the cells was measured using a NovoCyte 2000 flow cytometer (ACEA Biosciences, San Diego, CA, USA). The APC-MMR antibody-stained cells were excited using a 640-nm laser and detected using a 675 ± 30-nm bandpass filter. In all, 10,000 cells were counted for each sample, and the fluorescence intensity of the cell population was determined using Novo Express software (version 1.2.5, ACEA Biosciences).

### 3.8. Flow Cytometry Analysis of Intracellular Man-PRX Uptake

NIH/3T3 or RAW264.7 cells were plated in 24-well plates (5 × 10^4^ cells/well) and incubated overnight. The cells were then cultured in a treatment medium containing BODIPY-Man-PRX and BODIPY-HEE-PRX (10 μM PRX) at 37 °C for the prescribed durations. Subsequently, the cells were harvested, and their fluorescence intensity was measured using a NovoCyte 2000 flow cytometer. The BODIPY-labeled PRX-treated cells were excited using a 488-nm laser and detected using a 530 ± 30-nm bandpass filter. The fluorescence intensity of the treated cells was analyzed as described above.

### 3.9. Inhibition of Intracellular Man-PRX Uptake

RAW264.7 cells were plated in 24-well plates (5 × 10^4^ cells/well) and incubated overnight. The cells were then cultured in a medium containing different concentrations of α-d-mannose (Fujifilm Wako Pure Chemical) or anti-MMR antibody at 37 °C for 3 h. Next, the medium was replaced with a treatment medium containing BODIPY-Man-PRX (10 μM PRX), and the cells were cultured for 3 h at 37 °C. Lastly, the cells were harvested, and their fluorescence intensity was measured based on the description above.

### 3.10. CLSM Observation

RAW264.7 cells were plated in a 35-mm glass bottom dish (diameter of glass area: 12 mm) (Iwaki, Tokyo, Japan) at a density of 5 × 10^4^ cells/dish and incubated overnight. Next, the cells were cultured in a treatment medium containing BODIPY-Man-PRX or BODIPY-HEE-PRX (10 μM PRX) for 3 or 24 h. The cells were then stained with 500 nM LysoTracker Red DND-99 (Thermo Fisher Scientific) at 37 °C for 15 min, followed by staining with 1 μg/mL Hoechst 33,342 (Dojindo Laboratories, Kumamoto, Japan) at 37 °C for 10 min. CLSM observation was performed using FluoView FV10i (Olympus, Tokyo, Japan) equipped with a 60× water-immersion objective lens (numerical aperture 1.2). The excitation and emission wavelengths for Hoechst 33342, BODIPY-labeled PRXs, and LysoTracker Red were 405 nm and 455 nm, 473 nm and 520 nm, and 559 nm and 598 nm, respectively. The colocalization percentage was determined according to a previously reported method [46]. The values of the co-localization percentage are expressed as mean ± standard deviation of 100 cells.

### 3.11. Effect of RAW264.7 Cell Polarization on the Intracellular Man-PRX Uptake

RAW264.7 cells were plated in 24-well plates (5 × 10^4^ cells/well) and incubated overnight. The cells were then polarized for 24 h at 37 °C using 100 ng/mL LPS (Fujifilm Wako Pure Chemical) and 100 ng/mL recombinant mouse IFN-γ (PeproTech, Rocky Hill, NJ, USA) to produce the M1 phenotype, or 400 ng/mL recombinant mouse IL-4 (PeproTech) to produce the M2 phenotype. The expression level of MMR was determined as described above. Next, the medium was replaced with a treatment medium containing BODIPY-Man-PRX (10 μM PRX), and the cells were cultured for 3 h at 37 °C. Lastly, the cells were harvested, and their fluorescence intensity was measured as described above.

### 3.12. Quantitative RT-PCR

RAW264.7 cells were plated in six-well plates (1 × 10^6^ cells/well) and incubated overnight. Next, the cells were polarized as described above. Total RNA was isolated from the cells using the RNeasy Mini Kit (Qiagen, Valencia, CA, USA), and the cDNA was synthesized using the iScript Advanced cDNA Synthesis Kit (Bio-Rad, Hercules, CA, USA). Next, a reaction mixture containing cDNA (2 μL), forward and reverse primers (0.8 μL final concentration: 4 μM), and SsoAdvanced SYBR Green Supermix (Bio-Rad) (10 μL) was prepared, and its volume was adjusted to 20 μL using RNase/DNase-free water. Quantitative RT-PCR was performed using the CFX Connect Real Rime PCR System (Bio-Rad) with the following thermal cycling conditions: 45 cycles of denaturation at 95 °C for 5 s, annealing at 58 °C for 10 s, and extension at 72 °C for 10 s. The sequences of the primer sets were as follows: mouse TNF-α, forward: 5′-CAT CTT CTC AAA ATT CGA GTG ACA A-3′, reverse: 5′-TGG GAG TAG ACA AGG TAC AAC CC-3′; mouse IL-6, forward: 5′-GAG GAT ACC ACT CCC AAC AGA CC-3′, reverse: 5′-AAG TGC ATC ATC GTT GTT CAT ACA-3′; mouse Arg-1, forward: 5′-CAT TGG CTT GCG AGA CGT AGA C-3′, reverse: 5′-GCT GAA GGT CTC TTC CAT CAC C-3′ and β-actin forward: 5′-GGC TGT ATT CCC CTC CAT CG-3′, reverse: 5′-CCA GTT GGT AAC AAT GCC ATG T-3′. The gene expression levels were calculated using the ΔΔCt method and normalized using the expression level of the β-actin gene.

### 3.13. Statistical Analysis

Statistical analyses were performed using OriginPro 8 (OriginLab, Northampton, MA, USA). Data from more than three groups were compared using one-way analysis of variance (ANOVA) followed by the Tukey-Kramer multiple comparison test. Data of two groups were compared by using the Student’s *t*-test. For all the tests, *p* < 0.05 was considered to be statistically significant. 

## 4. Conclusions

In the present study, we synthesized α-d-mannose-modified α-CD/PEG-based PRX, and examined its intracellular uptake in macrophage-like RAW264.7 cells. Intracellular Man-PRX uptake was observed in MMR-positive RAW264.7 cells but was negligible in the MMR-negative NIH/3T3 cells. In addition, the intracellular Man-PRX uptake in RAW264.7 cells was significantly decreased in the presence of free α-d-mannose and anti-MMR antibody. These results suggest that Man-PRX was internalized in RAW264.7 cells specifically through MMR recognition. Moreover, our results showed that the polarization of RAW264.7 cells affected the internalization efficiency of Man-PRX. In addition, our results showed that Man-PRX was preferentially taken up by M2 macrophages that showed a high MMR expression level. According to these results, the modification of PRX using α-d-mannose is an effective method for selective targeting of macrophages. Man-PRX contributes to the treatment and diagnosis of various diseases, including metabolic disorders.

## Abbreviations

PRX	Polyrotaxane
CD	Cyclodextrin
MMR	Macrophage mannose receptor
Man-PRX	α-d-Mannose-modified polyrotaxane
HEE-PRX	2-(2-Hydroxyethoxy)ethyl carbamate-modified polyrotaxane
PEG	Poly(ethylene glycol)
CDI	1,1′-Carbonyldiimidazole
DMSO	Dimethyl sulfoxide
Man-NH_2_	2-Aminoethyl α-d-mannopyranoside
DBU	1,8-Diazabicyclo[5.4.0]undec-7-ene
SEC	Size exclusion chromatography
FT-IR	Fourier transform infrared
NMR	Nuclear magnetic resonance
SPR	Surface plasmon resonance
APC	Allophycocyanin
CLSM	Confocal laser scanning microscopy
LPS	Lipopolysaccharide
IL	Interleukin
IFN-γ	Interferon γ
TNF-α	Tumor necrosis factor α
Arg-1	Arginase-1
MWCO	Molecular weight cut-off
HEPES	2-[4-(2-Hydroxyethyl)piperazin-1-yl]ethanesulfonic acid
DMEM	Dulbecco’s modified Eagle’s medium.
